# Cochlear Implantation in Noonan Syndrome With and Without Multiple Lentigines: A Case Report and Systematic Review

**DOI:** 10.1097/ONO.0000000000000009

**Published:** 2022-03-08

**Authors:** Daniel Blumenthal, Braeden Lovett, James Leonard, Sixian Wang, Melissa Blumgart, Michael Hoa

**Affiliations:** 1 Department of Otolaryngology-Head & Neck Surgery, Georgetown University Medical Center, Washington, DC; 2 Georgetown University School of Medicine, Washington, DC.

**Keywords:** Cochlear implant, Hearing loss, Noonan syndrome with multiple lentigines, *PTPN11*, Sensorineural hearing loss

## Abstract

**Objectives:**

To describe outcomes after bilateral cochlear implantation (CI) in a patient with a pathologic *PTPN11* variant associated with Noonan syndrome (NS) and Noonan syndrome with multiple lentigines (NSML). Additionally, to assess the utility of CI in this specific population based on our outcome and previous reports.

**Study Design:**

Retrospective case report with literature review using Preferred Reporting Items for Systematic Reviews and Meta-Analyses guidelines.

**Patients:**

A young boy with various multiorgan abnormalities, speech and language delay, and persistent hearing loss who was found to have a heterozygous *PTPN11* gene mutation at age 2.

**Interventions:**

Bilateral tympanostomy tube placement, diagnostic imaging, and eventual staged bilateral CI.

**Main Outcome Measures:**

Objective audiometric testing and developmental milestone attainment.

**Results:**

Bilateral CI was successfully completed over a 2-month period. The patient illustrated significant improvement in objective audiologic measurement. However, he continues to sign as his main form of communication without significant speech progression.

**Conclusions:**

Early diagnostic and therapeutic intervention in patients with NS/NSML can help improve long-term audiologic and speech development. Given the heterogeneity of NS/NSML, a multidisciplinary approach is needed for optimal outcomes.

Noonan syndrome with multiple lentigines (NSML), previously known as LEOPARD syndrome, is an autosomal dominant disorder most commonly caused by a mutation in the tyrosine phosphatase non-receptor type 11 gene (*PTPN11*) (1). This gene is also involved in Noonan syndrome (NS), which occurs in 1 in 1000 to 2500 live births (1).

NSML and NS share a broad range of clinical findings including lentigines, electrocardiographic conduction defects, hypertelorism, pulmonary stenosis, genital abnormalities, deafness, and growth and mental retardation (2). Due to phenotypic variation, clinical diagnosis is challenging and often supplemented with genetic testing.

There are a number of studies reporting hearing loss (HL) in both NS and NSML patients. Sharland et al (3) described HL in 58 out of 146 NS patients, 3% of which suffered from isolated sensorineural hearing loss (SNHL). van Trier noted HL in 34 of 97 NS patients. SNHL was present in 20% of these patients, while mixed HL and conductive HL were each found in 4% (4). Other studies demonstrate various temporal bone abnormalities in patients with NS/NSML (5,6).

Despite the prevalence of HL in both NS and NSML patients, outcomes after cochlear implantation (CI) in this population remain poorly characterized. This case and systematic review assess the literature regarding CI outcomes in NS and NSML patients, illustrate our experience with bilateral CI in a patient with a *PTPN11* mutation, and further explore the genetics behind these unique syndromes.

## MATERIALS AND METHODS

A case report of a patient with a *PTPN11* mutation undergoing bilateral CI is described. A systematic review was performed, which included case reports/series of patients with NS and/or NSML who underwent CI, without restriction for gender or age. Articles of any publication date and status were accepted. Exclusion criteria included reviews, editorials, commentaries and papers published in a non-English language. Studies were identified in ovid Medline and PubMed using the keywords: leopard syndrome, cochlear implantation, noonan syndrome, and hearing loss. Reference lists of retrieved review papers were manually searched to identify additional relevant studies. The last search was performed on March 30, 2021. No review protocol was used in this review.

Eligibility was assessed by 3 independent investigators (D.B., B.L., J.L.) and disagreements were resolved by consensus. After the initial literature search, each study was screened for eligibility based on title and abstract. Full text was retrieved for all articles that met inclusion criteria and independently reviewed by the authors (D.B., B.L., J.L.). Data extracted included: demographics (eg, age, gender, ethnicity), diagnosis and genetic variant if available, pre- and post-CI audiology results, diagnostic imaging, and follow-up assessments. Bias risk assessment tools were not employed given the nature of included articles (ie, case reports/series). The authors (D.B., B.L.) graded the quality of evidence for each study using the modified version of the Oxford Centre for Evidence-Based Medicine levels of evidence. Each study included was a case report or case series; therefore, level 4 evidence.

## RESULTS

**FIG. 1.**
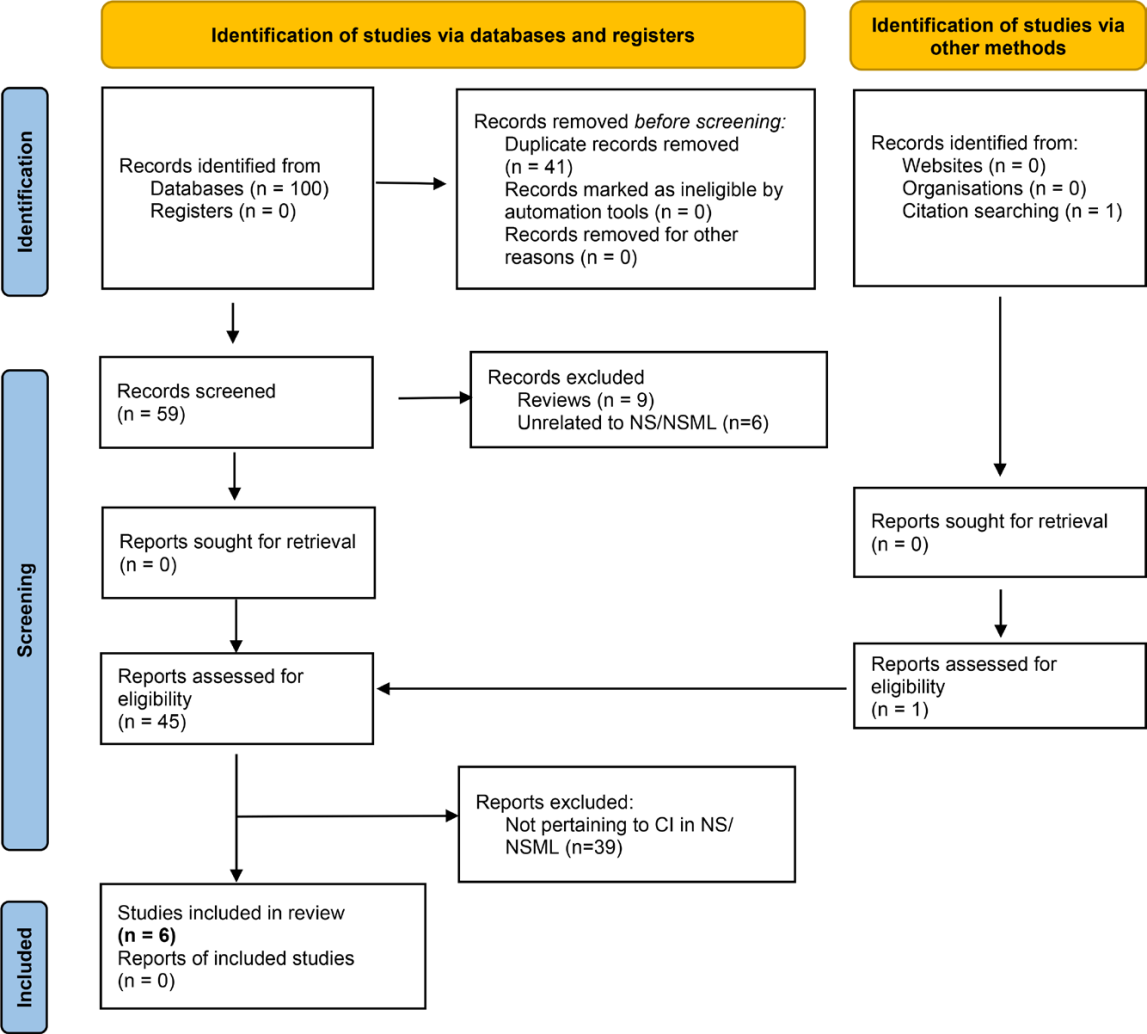
PRISMA workflow. From: Page MJ, McKenzie JE, Bossuyt PM, et al. The PRISMA 2020 statement: an updated guideline for reporting systematic reviews. *BMJ* 2021;372:n71. doi: 10.1136/bmj.n71. CI indicates cochlear implantation; NS, Noonan syndrome; NSML, Noonan syndrome with multiple lentigines; PRISMA, Preferred Reporting Items for Systematic Reviews and Meta-Analyses.

Literature and manual search of reference lists yielded a total 60 articles after duplicate removal. After title and/or abstract screening, 9 review articles were excluded, as well as 6 articles for not pertaining to patients with NS/NSML. Of the remaining 45 articles, 6 met inclusion criteria and were included in this review (Fig. 1). These 6 articles are organized in Table 1.

**TABLE 1.** Systematic review of NS and/or NSML patients undergoing cochlear implantation

Study	Subject	Ear findings	Other findings	Preoperative audiometry	Imaging/perioperative findings	Postoperative outcomes	Level of evidence^*a*^
Satar et al (6)	NS, male age 3 y	Anomalous pinnae	NA	NA	Absent superior/lateral SCs, horizontal SC bud, absent vestibular aqueduct and small oval window (R)	NA	4
Scheiber et al (7)	NS, female	Low-set, posteriorly angulated, thickened helix	Short stature, pterygium colli, hypertelorism, ptosis, epicanthus medialis, high anterior/low posterior hairline, high/wide nasal bridge, deep philtrum	ABR: 100 dB (R), 95 dB (L)	No inner ear abnormalities	PTA: 25 dB	4
	(c.922A>G)			Flat tympanograms		Encouraging progress in speech and general development	
	Age 4 y						
				+ EAMFRs			
	NS, female	Low-set, posteriorly angulated, thickened helix	Short stature, triangular facial contour, short webbed neck with pterygium colli, hypertelorism, ptosis, epicanthus medialis, high anterior hairline, high nasal bridge, wide nasal base, deep philtrum	ABR: no response	No inner ear abnormalities	PTA: 45 dB (R)	4
	(c.853T>C)			< 60 dB BC, 100 dB AC		Encouraging progress in speech and general development	
	Age 2 y			ABR: 100 dB (R), 100 dB (L)			
				Flat tympanograms			
				+ EAMFRs			
Chu et al (8)	NSML, female	Low-set	Hypertelorism, plane occiput	ABR: none (B/L)	Enlarged B/L vestibular aqueducts	At 1 y: CAP 1	4
	(c.1381G>A)			ASSR: 120 dB at 1 kHz, 115 dB at 2 kHz (L), 125 dB at 4 kHz (R)		Able to respond to loud sound but no meaningful communication	
	Age 5 (L) y						
Vermeire et al (9)	NSML, male age	NA	NA	IT-MAIS 12/40	NA	At 1.5 y	4
	1 (R)–1.5 (L) y			Aided PTA: 50–80 dB		PTA: 17.5 dB	
						CAP 5	
						IT-MAIS 37/40	
						SiR category 4	
						Doing well in regular preschool program with a teacher of the deaf and home-based speech therapy	
van Nierop et al (10)	NS, male	No inner ear abnormalities	Downward slanting palpebral fissures, dysmorphic face, systolic heart murmur	Vestibular hyporeflexia via electronystagmography	No inner ear abnormalities	At 7 mo: Soundfield PTA: 35 dB (R)	4
	(c.1510A>G)			Aided ASSR: 90 dB (R), 75 dB (L)		At 3 y: phoneme score 63%	
	Age 1 y (R)			Aided VRA: 53 dB (R), 43 dB (L)		Slow progress in verbal language development attributed to intellectual disability and environment with sign language	
	NS, male	Low-set, dysplastic	Short stature, erythroderma, hypertelorism, sandal-gap deformity, pterygium colli, retrognathia, xerosis cutis	Asymmetric vestibular areflexia	No inner ear abnormalities on MRI	At 12 mo: PTA 43 dB (R)	4
	(c.124A>G)			Aided ASSR: 105 dB (R), 93 dB (L)	Posteriorly displaced chorda tympani	At 36 m: PTA 28 dB (R)	
	Age 1.7 y (R)			Aided VRA: 80 dB		At 7 y: phoneme score 90%	
						Slow improvement in language comprehension attributed to intellectual disability	
	NS, female	Cup ear (R), dysplastic helix (L)	Short stature, ptosis, epicanthus, systolic murmur	B/L vestibular areflexia	No inner ear abnormalities	At 2 mo: ASSR PTA: 45 dB	4
	(c.922A>G)			Aided soundfield PTA: 70 dB		At 6 y: PTA: 28 dB, phoneme score 70%	
	Age 3.75 y (R)					Obvious progress seen in language development though persistent delay noted	
	NS, female	Low-set	Severe pectus excavatum, short stature and neck	Aided PTA: 113 dB (R), 115 dB (L)	NA	At 7 mo: PTA: 22 dB, phoneme score 87%	4
	(c.124A>G)						
	Age 13.83 y (R)						
				Phoneme score 25% (R), 44% (L)		Persistent delayed language development	
	NSML, female	B/L mastoid opacification	Short stature, systolic murmur, frontal bossing, mild ptosis, large café-au-lait spot on buttocks, multiple lentigines	ABR: no response at 90 dB	B/L mastoid opacification	At 1 y: PTA: 32 dB (R), 25 dB (L); phoneme score 85% (R), 60% (L)	4
	(c.836A>G)			Aided PTA: 40 dB		At 2–3 y: phoneme score 90% (R), 89% (L)	
	Ages 1.33 (R)–1.83 (L) y					Language comprehension became age-appropriate	
van Trier DC et al (4)	4 subjects NS	NA	NA	NA	NA	NA	4

^*a*^OCEBM Levels of Evidence Working Group*. “The Oxford Levels of Evidence 2.” Oxford Centre for Evidence-Based Medicine. https://www.cebm.ox.ac.uk/resources/levels-of-evidence/ocebm-levels-of-evidence.

ABR indicates auditory brainstem response; AC, air conduction; ASSR, auditory steady-state response; B/L, bilateral; BC, bone conduction; CAP, central auditory processing test; EAMFR, electrically evoked amplitude modulation following response; IT-MAIS, infant-toddler meaningful auditory integration scale; L, left; NA, not available; NS, Noonan syndrome; NSML, Noonan syndrome with multiple lentigines; PTA, pure-tone average; R, right; SC, semicircular canal; SiR, speech intelligibility rating; VRA, visual reinforcement audiology; y; year.

### Case

A full term male presented with notable speech and language delay at age 15 months. Gestation and delivery were uncomplicated and family history was unremarkable for syndromic pathology. No abnormalities were identified at birth and the patient passed his newborn hearing screening. During his first year of life, he was diagnosed with pulmonary valve stenosis, otitis media with effusion, and bilateral nephrosis. At presentation, audiologic evaluation revealed absent transient evoked otoacoustic emissions (TEOAEs) and flat tympanometry on the right, and unattainable TEOAEs with normal tympanometry on the left. Repeat testing at age 2 illustrated type B tympanograms bilaterally and mild-to-moderate bilateral HL at 500–1000 and 4000 Hz. Minimal improvement was noted after bilateral myringotomy and tympanotomy tube placement. Subsequently, sedated audiometry brainstem response revealed bilateral severe-to-profound SNHL from 500 to 4000 Hz.

**FIG. 2.**
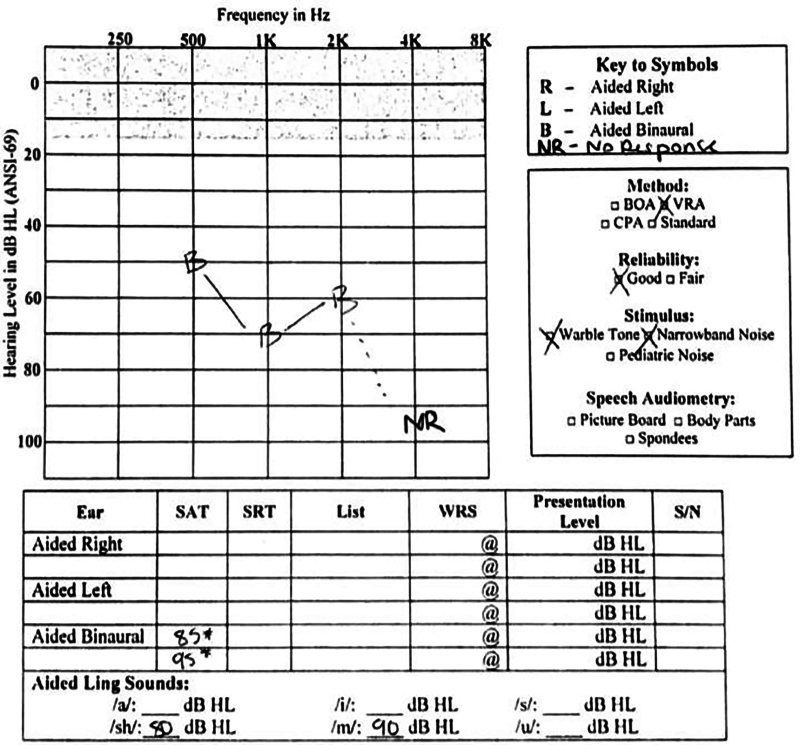
Preoperative binaural aided soundfield audiogram. Moderate SNHL at 500, 1K, and 2K Hz. No response at 4K or 5K Hz. Binaural SAT 85–95 dB. ANSI-69 indicates American National Standards Institute-69 (refers to a device number designation); BOA, behavioral observation audiometry; CPA, conditioned play audiometry; HL, hearing loss; SAT, speech awareness threshold; S/N, signal to noise ratio; SNHL, sensorineural hearing loss; SRT, speech reception threshold; VRA, visual reinforcement audiology; WRS, word recognition score.

The patient failed to make progress after a 6-week trial with bilateral hearing aids. Subsequent audiometry illustrated response to tonal and narrowband noise stimuli in the moderate-to-severe hearing range from 500 to 2000 Hz, but absent response at 4000 Hz (Fig. 2). Both speech awareness threshold (SAT) and Aided Ling Sounds were found in the severe-to-profound HL range. As a result, bilateral CI was recommended. Meanwhile, the patient underwent genetic testing given the presence of SNHL and pulmonary valve stenosis, revealing a heterozygous *PTPN11* gene with a c.1529A>G variant.

**FIG. 3.**
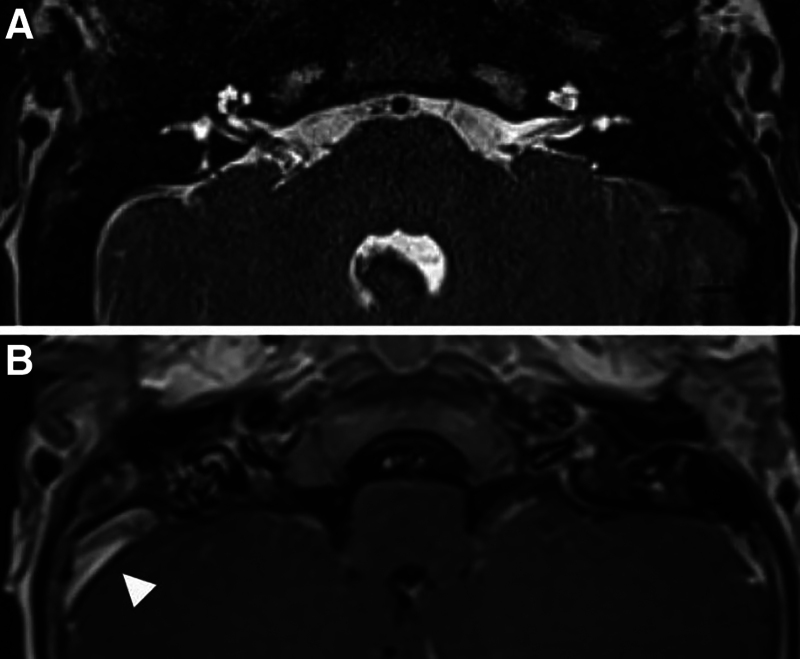
MRI of the IAC. *A*, constructive interference in steady state axial image demonstrating normal-appearing cochlea and inner ears bilaterally. *B*, T1 axial post-contrast showing anterior and dominant sigmoid sinus on the right (arrow). IAC indicates internal auditory canal.

Preoperative MRI of the internal auditory canal demonstrated normal-appearing inner ear structures with no evidence of retrocochlear lesions (Fig. 3). At 2.5 years of age, CI was performed successfully in the left ear but postponed in the right due to poor insertion trajectory secondary to anterior displacement of the facial nerve. There was also notable round window ossification on the right side. Right ear CI was reattempted 2 months later, which required incus buttress removal and intraoperative CT-based guidance. Full electrode insertions were achieved with each surgery.

**FIG. 4.**
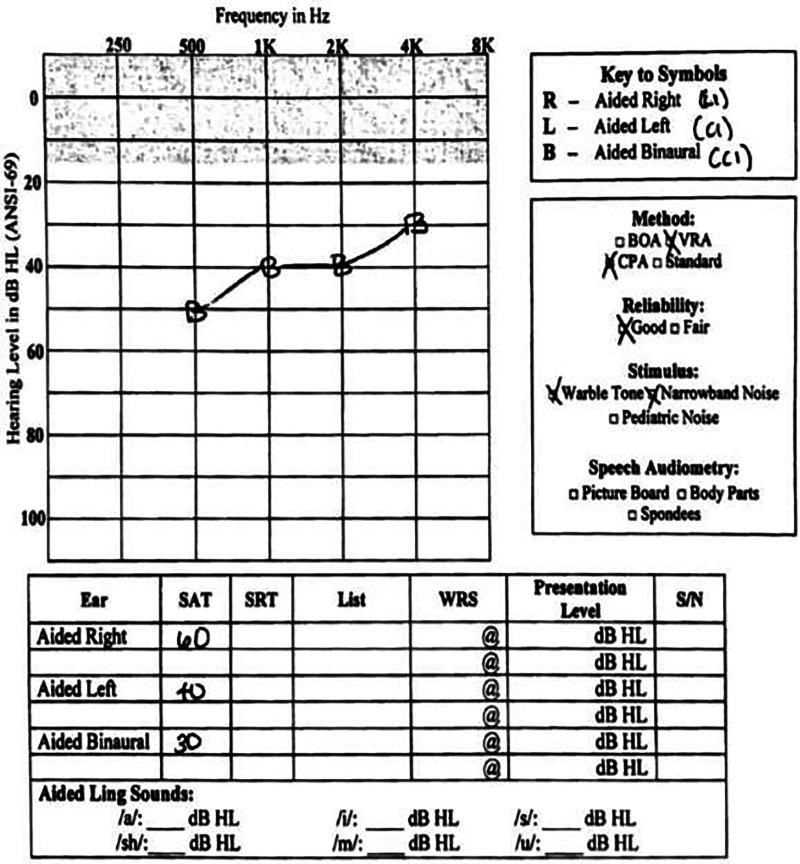
At 7 weeks post right CI and 5 weeks post left CI. Moderate SNHL at 500, 1K, and 2K Hz. Mild hearing loss at 4K. Binaural SAT 30 dB. ANSI-69 indicates American National Standards Institute-69 (refers to a device number designation); BOA, behavioral observation audiometry; CI, cochlear implantation; CPA, conditioned play audiometry; HL, hearing loss; SAT, speech awareness threshold; S/N, signal to noise ratio; SNHL, sensorineural hearing loss; SRT, speech reception threshold; VRA, visual reinforcement audiology; WRS, word recognition score.

Audiogram at 7 months after activation of his left CI and 5 months after activation of right CI is shown (Fig. 4). Audiologic testing revealed an SAT of 40 dB HL on the right, left, and bilaterally. The patient is now able to consistent discern speech and sounds. However, the patient continues to predominantly communicate through sign.

## DISCUSSION

Individuals with NS and NSML possess variable and overlapping phenotypic characteristics; including diverse otologic manifestations and hearing abnormalities. Our experience adds to the growing list of audiologic abnormalities found in patients with pathologic *PTPN11* variants. The patient’s 7-month postoperative outcomes advocate for early intervention with CI for improved audiometric function. However, practitioners and parents must be aware of the mixed speech outcomes seen in patients with underlying cognitive deficits.

CI was surgically successful in our patient and in all documented cases despite anatomical challenges. All accounts in the literature that included preoperative and postoperative audiometric measures displayed marked improvement in auditory performance over time with differing speech and language outcomes. van Nierop et al (10) describe 5 patients with improved audiometric measures and phoneme discrimination testing; however, 4 patients continued to have delayed speech and language development. Similarly, Scheiber et al (7) describe 2 patients with improved objective audiologic findings and subsequent scholastic improvement through educational milestones. Vermeire et al (9) reported a 1-year-old with bilateral SNHL who acclimated to a regular school program with home speech therapy 1.5 years later after CI. In another study, a 5-year-old with bilateral profound SNHL showed improvement in hearing perception 1 year after CI, but was unable to achieve meaningful communication (8).

Our patient’s auditory thresholds demonstrate objective improvement in his ability to detect sounds after CI. However, his speech development remains limited despite intensive auditory verbal therapy and immersion in a learning environment with an emphasis on auditory and verbal communication. His course illustrates the uncertain outcomes in NS/NSML patients secondary to their underlying intellectual deficits. Long-term follow-up with Otolaryngology and speech language pathology practitioners is vital to help children with similar impairments progress past sign communication. Additionally, parents must be made aware that outcomes are difficult to predict and that successful CI placement and audiometric improvement may not translate quickly, if at all, to oral speech.

Mental deficiencies in various domains have been reported in NS/NSML patients. These include poor attention and executive functioning, diminished verbal skills and reasoning, and special educational requirements (11–13). Roelofs et al (12) found that children with NS have low performance and verbal intelligence quotient (IQ) while adults advance to a normal performance IQ over time. However, verbal IQ does not develop proportionately. This longitudinal study does not include patients with CI; however, it does provide important information that helps inform parents and patients on long-term expectations.

Although HL and timely interventions play a role, it is possible that *PTPN11* gene variants impact cognitive development. Notably, Gauthier et al (14) demonstrated the critical role of *PTPN11* gene products in neurogenesis and regulation using in vitro murine cultured cortical precursors. The in vivo cortical abnormalities observed may help to explain the cognitive impairment resistant to improved audiologic measures and the varied outcomes after CI. More research is required to better identify specific mutations that are associated with treatment-resistant behavioral delay.

The large variability in clinical manifestations and outcomes in NS/NSML is related to the genetic heterogeneity of these overlapping syndromes. *PTPN11* gene mutations are most commonly seen in inherited forms of NS (50%) and NSML (>85%), but other variants have been implicated (15). A number of *PTPN11* variants also exist, and evidence illustrates inconsistent phenotypic presentation among patients with the same mutation (16). This is evident in 2 patients reviewed with the c.992A alteration who underwent CI. One patient passed newborn hearing screening while the other failed. This not only denotes inconsistent phenotypic expression but suggests that newborn hearing screens may not rule out HL in NS and NSML patients (7,10).

Interestingly, gene expression studies localize *PTPN11* to hair cells, supporting cells and spiral ganglion neurons (SGNs). However, they are predominantly found in SGN over the other 2 locations (17). Shearer et al (17) illustrate that mutations specific to the SGNs are the most predictive factors for CI failure to date and may explain the poor outcomes in NS and NSML patients. There are numerous documented SGN-specific mutations including but not limited to *TMPRSS3*, *AIFM1*, *DIAPH3*, *DFNB59*, *MT-RNR1*, and *OPA1*. These discoveries bring with them a growing concern that patients with these defects may not gain the same benefit from CI as those with defects in other areas of the peripheral auditory system (17,18). However, there is limited data in SGN-specific mutations’ CI outcomes due in large part to the low overall incidence and novel area of research. Moving forward, genetic testing should be encouraged prior to CI to allow practitioners and parents to engage in a more informative discussion regarding prognosis following CI based on the location of their child’s mutation.

The genetic complexity behind clinical outcomes, however, must be weighed conjointly with the benefits of early intervention. Holder et al (19) recently reported on improved CI outcomes in pediatric patients with *TMPRSS3* mutations when compared to their adult counterparts undergoing the same procedure later in life. The specific mutation is found in SGNs and is thought to cause SNHL. These authors advocate for early intervention in their pediatric population. Whether a similar or earlier window of opportunity exists in patients with *PTPN11* mutations is unknown. While the mutations differ, this study reinforces that early CI implantation may benefit patients with *PTPN11* mutations despite their underlying deficits. More widespread and early genetic testing may identify individuals with these mutations prior to the onset of HL and enable a better clinical monitoring for disease progression.

## CONCLUSIONS

Given the heterogeneity of NS/NSML, a multidisciplinary approach is needed for optimal outcomes. Early genetic testing in patients with SNHL and multivisceral abnormalities is critical for diagnosis, patient education, prognosis, and joint decision-making. Although there is limited data on CI outcomes in NS/NSML patients, this intervention should be discussed with parents as a viable treatment modality to improve audiologic and speech development. Identifying the specific mutation remains an underutilized modality and should be incorporated into practice better counsel patients on their audiologic and speech improvements after CI placement. Future studies that stratify cognitive deficits based on specific mutations will enhance our understanding of the pathophysiology behind NS/NSML and improve clinical decision-making when predicting CI outcomes.

## FUNDING SOURCES

None declared.

## CONFLICT OF INTEREST

M.H. holds the position of Associate Editor for Otology and Neurotology Open and has been recused from reviewing or making decisions for the article. The remaining author discloses no conflicts of interest.

## DATA AVAILABILITY

All data gathered and analyzed from systematic review is provided in this study. All relevant deidentified clinical data is provided in this manuscript.
