# Cochlear Implantation in Noonan Syndrome With and Without Multiple Lentigines: A Case Report and Systematic Review: Erratum

**DOI:** 10.1097/ONO.0000000000000031

**Published:** 2023-03-23

In the following article published in *Otology & Neurotology Open,* while informed consent was documented prior to acceptance of the article, this information was erroneously omitted from the final publication.

*Otology & Neurotology Open*. 2(1):e009, March 2022.

Cochlear Implantation in Noonan Syndrome With and Without Multiple Lentigines: A Case Report and Systematic Review

https://journals.lww.com/onojournal/Fulltext/2022/03000/Cochlear_Implantation_in_Noonan_Syndrome_With_and.2.aspx
